# Effects of hemicellulose on intestinal mucosal barrier integrity, gut microbiota, and metabolomics in a mouse model of type 2 diabetes mellitus

**DOI:** 10.3389/fmicb.2023.1096471

**Published:** 2023-02-06

**Authors:** Huan Liu, Jihao Xu, Chiuwing Yeung, Qikui Chen, Jieyao Li

**Affiliations:** Department of Gastroenterology, Sun Yat-sen Memorial Hospital, Sun Yat-sen University, Guangzhou, Guangdong, China

**Keywords:** gut microbiota, gut metabolome, T2DM, hemicellulose, gut barrier damage

## Abstract

**Background and objective:**

Impaired gut barrier contributes to the progression of type 2 diabetes mellitus (T2DM), and the gut microbiota and metabolome play an important role in it. Hemicellulose, a potential prebiotics, how its supplementation impacted the glucose level, the impaired gut barrier, and the gut microbiota and metabolome in T2DM remained unclear.

**Methods:**

In this study, some mice were arranged randomly into four groups: db/db mice fed by a compositionally defined diet (CDD), db/db mice fed by a CDD with 10% and 20% hemicellulose supplementation, and control mice fed by a CDD. Body weight and fasting blood glucose levels were monitored weekly. The gut barrier was evaluated. Fresh stool samples were analyzed using metagenomic sequencing and liquid chromatography-mass spectrometry to detect gut microbiota and metabolome changes. Systemic and colonic inflammation were evaluated.

**Results:**

Better glycemic control, restoration of the impaired gut barrier, and lowered systemic inflammation levels were observed in db/db mice with the supplementation of 10 or 20% hemicellulose. The gut microbiota showed significant differences in beta diversity among the four groups. Fifteen genera with differential relative abundances and 59 significantly different metabolites were found. In the db/db group, hemicellulose eliminated the redundant *Faecalibaculum* and *Enterorhabdus*. The increased succinate and ursodeoxycholic acid (UDCA) after hemicellulose treatment were negatively correlated with *Bifidobacterium, Erysipelatoclostridium*, and *Faecalibaculum*. In addition, hemicellulose reduced the colonic expressions of TLR2/4 and TNF-α in db/db mice.

**Conclusion:**

Hemicellulose may serve as a potential therapeutic intervention for T2DM by improving impaired intestinal mucosal barrier integrity, modulating gut microbiota composition, and altering the metabolic profile.

## Highlight

- The impact of hemicellulose on gut barrier integrity, gut microbiota composition, and metabolic profile in a mice model with type 2 diabetes mellitus (T2DM) was investigated. The results indicated that treatment with hemicellulose may have a beneficial effect on gut barrier function and the gut microbiome and metabolome in T2DM.

## Introduction

The prevalence of type 2 diabetes mellitus (T2DM) continues to increase worldwide (Federation, 2019). T2DM-related complications affect various organs and systems, including the intestine. Our previous studies showed that impaired intestinal barriers and higher gut permeability existed in both patients with T2DM and mouse models (Cheng et al., 2018; Yuan et al., 2021). The tight epithelial junction (TJ) was one of the crucial aspects of the gut barrier, which could regulate gut permeability and the entry of intestinal contents. It was acknowledged that chronic systemic inflammation states have a great impact on the progression of T2DM (Donath and Shoelson, 2011). The destruction of the TJ could lead to the translocation of the gut bacteria and their pathogen-associated molecular patterns (PAMPs), which exacerbated systemic inflammation and, in turn, contributed to the progression of T2DM (Ghosh et al., 2020; Yang et al., 2021). Ameliorating the impairment of the intestinal mucosal barrier might be a potential way to help control T2DM.

However, studies showed that individuals with T2DM and T2DM mouse models exhibit alterations in gut microbiota composition when compared to non-DM controls (Qin et al., 2012; Zhang et al., 2019). These changes, also known as gut dysbiosis, lead to increased intestinal inflammation, impairments in intestinal barrier, and higher gut permeability (de Vos et al., 2022). It was demonstrated that consumption of dietary fiber could counteract gut dysbiosis, which might, in turn, ameliorate the impaired gut barrier (Guo et al., 2021; Wlodarczyk and Slizewska, 2021). Dietary fibers, such as hemicellulose, have been proposed as potential prebiotics. However, the effect of hemicellulose on T2DM-associated disorders of the intestinal mucosal barrier, gut microbiota, metabolome, and glycemic control is not well understood. Therefore, the aim of this study is to investigate these effects.

## Materials and methods

The present study was conducted in accordance with guidelines set by the Animal Ethics Committee of Sun Yat-sen University (permit code: SYSU-IACUC-2021-000391) and adhered to the ARRIVE guidelines for animal research.

### Experimental design

Male BKS.Cg-Dock7m +/+ Leprdb/JNju (db/db) mice and male C57BL/6 mice were purchased from Guangdong Yaokang Biotechnology Co., Ltd. db/db mice were used as spontaneous T2DM models. C57BL/6 mice were used as a control to evaluate the pathologic changes caused by T2DM. Male mice were used to reduce the impact of sex differences on T2DM. All the mice were between 4 and 6 weeks old. Mice were fed *ad libitum* in a 12-h daylight cycle. The housing temperature was 24 ± 2°C, and the relative humidity was 52 ± 8%. Hemicellulose was purchased from the Guangxi Institute of Botany, Chinese Academy of Sciences. The diet was synthesized using Dayz Biotechnology Co., Ltd. (China). In line with previous studies, a compositionally defined diet (CDD) was used as a control diet with a total fiber content of only about 5% (Desai et al., 2016; Zou et al., 2018). The diet's overall fat, protein, and carbohydrate levels supplemented with hemicellulose mimicked those of CDD. The ingredient composition of the experimental diets is shown in Table 1 after 1 week of acclimatization with CDD. The db/db mice were randomly arranged into three groups (*n* = 5–7/group): the db/db group fed with CDD; the 10%-hemi group fed with CDD supplemented with 10% (w/w) hemicellulose; and the 20%-hemi group fed with CDD supplemented with 20% (w/w) hemicellulose. Moreover, the C57BL/6 mice were assigned to the control group fed with CDD. These four groups were defined as the A, B, C, and D groups, respectively. The body weight was monitored once a week during the trial period. The ONE TOUCH UltraEasy glucometer (1955,685, Johnson and Johnson, USA) was used to check fasting blood glucose levels one time a week at 4:00 PM after an 8-h fast.

**Table 1:** Composition of the experimental diets.

**Ingredient (g)**	**Compositionally defined diet**	**10%-hemi**	**20%-hemi**
Caisein	200	200	200
L-Cystine	3	3	3
Sucrose	68.8	68.8	68.8
Dyetrose	125	125	125
Cornstarch	506.2	506.2	506.2
Lard	20	20	20
Soybean Oil	25	25	25
Cellulose	50	50	50
Hemicellulose	0	117.23	263.76
Mineral mix	10	10	10
Calcium carbonate	5.5	5.5	5.5
Dicalcium phosphate	13	13	13
Potassium citrate	16.5	16.5	16.5
Vitamin mix	10	10	10
Choline bitartrate	2	2	2
Dye	0.05	0.05	0.05
Total	1,055.05	1,172.28	1,318.81

### Sample collection

After 8 weeks, the cervical vertebrae of the mice were dislocated after CO_2_ anesthesia, and the abdomen was opened. The colon contents were collected and stored at −80°C for the subsequent metagenomic sequencing and metabolomic analysis. Colon segments were immersed in glutaraldehyde for transmission electron microscopy analysis. Others were immediately transferred to the −80°C freezer for further analysis.

### Total RNA extraction and quantitative real-time PCR (qRT-PCR)

Total RNA extraction from the colon tissue was performed using the TRIzol reagent (Takara, Japan). The concentration was calculated by the absorbance at 260 nm using the Nanodrop2000 (NanoDrop Technologies, USA). The PrimeScriptTM RT reagent Kit (Takara, Japan) was used to synthesize cDNA from 500 ng of total RNA. qRT-PCR was performed with SYBR^®^ Premix Ex Taq™ II (Takara, Japan) on a Bio-Rad Real-Time PCR instrument (Bio-Rad, USA). The primers are shown in Table 2. Data were analyzed using the 2^−ΔΔCt^ method, with β-actin as an internal control (Bio-Rad CFX Manager 2.1, USA).

**Table 2:** The primer sequences for qPCR.

**Gene**	**Forward primers**	**Reverse primers**
Occludin	TTTCCTGCGGTGACTTCTCC	GGGGAACGTGGCCGATATAA
ZO-1	GACTTGTCAGCTCAGCCAGT	GGCTCCTCTCTTGCCAACTT
TLR2	GAGCATCCG AATTGCATCACC	CCCAGAAGCATCACATGACAGAG
TLR4	GAGCCGGAAGGTTATTGTGGT	CCTCTGCTGTTTGCTCAGGAT
TNF-α	CCCTCCAGAAAAGACACCATG	CACCCCGAAGTTCAGTAGACAG
β-actin	GTCATCACTATTGGCAACGAGC	TACGGATGTCAACGTCACACTT

### Western blotting assay

Samples from the colon were lysed with RIPA lysis buffer (CW Biotech, China) and PMSF (CW Biotech, China). Equal amounts of total proteins (20 mg per lane) were separated by SDS-PAGE (Millipore, USA). Consequently, 5% BSA was used to block the membrane for 2 h at room temperature. Then, the protein bands were incubated with the primary antibodies ZO-1 (1:1,000, CST, USA), Occludin (1:1,000, CST, USA), and β-actin (1:2,000, Santa Cruz, USA) overnight at 4°C. The membranes were incubated with goat anti-mouse IgG-HRP (1:5,000, CWBiotech, China) or goat anti-rabbit IgG-HRP (1:5,000, CWBiotech, China) for 1 h. Proteins were detected by enhanced chemiluminescence. Loading differences were normalized using the constitutive marker, β-actin.

### Transmission electron microscopy (TEM)

Colonic tissue samples were cut into 1mm^3^ sections on ice and fixed overnight at 4°C with 2.5% glutaraldehyde and 2.0% paraformaldehyde. Afterward, samples were washed with 0.1 mol/L phosphate-buffered saline (PBS) and post-fixed with 1% osmic acid for 2 h at 4°C. Then, the samples were dehydrated in graded acetone and embedded with Epon 812. The sections were cut into semi-thin pieces and double-stained with 2.0% uranyl acetate and 2.0% lead citrate. TEM (JEM-100CXII; JEOL, Japan) with an accelerating voltage of 100 kV was used to observe and photograph the colonic epithelial tight junction ultrastructure.

### Lipopolysaccharide (LPS) measurement

Serum LPS quantification was performed using a commercial kit with a *Limulus* amebocyte extract (Lal kit; Cambrex BioScience, USA).

### Colonic microbiota analysis

#### Extraction of genome DNA

Genomic DNA was extracted using a commercial kit according to the manufacturer's instructions [ALFA Stool DNA Extraction Mini Kit (FINDROP, Guangzhou)]. DNA integrity and purity were monitored on 1% agarose gels. DNA concentration and purity were measured using Qubit 3.0 (Thermo Fisher Scientific, Waltham, USA) and Nanodrop One (Thermo Fisher Scientific, Waltham, USA) at the same time.

#### Library preparation and sequencing

Sequencing libraries were generated using the ALFA-SEQ DNA Library Prep Kit for Illumina (FINDROP, Guangzhou) following the manufacturer's protocols, and index codes were added. The library quality was assessed on the Qubit 4.0 Fluorometer (Life Technologies, Grand Island, NY) and the Qsep400 High-Throughput Nucleic Acid Protein Analysis System (Houze Biological Technology Co., Hangzhou, China). Finally, the library was sequenced on an Illumina NovaSeq 6,000 platform, and 150-bp paired-end reads were generated. The sequencing was conducted through Guangzhou Yike Biotechnology Co., Ltd. (Guangzhou, China).

#### Data analysis

Trimmomactic software (LEADING:3 TRAILING:3 SLIDINGWINDOW:5:20 MINLEN:50) was used for quality control the raw data to remove low-quality bases and reads. Software MEGAHIT was used for the mixed assembly with all reads. We obtained the scaftigs by separating the mixed-assembly scaffolds from the *N* connection. We filtered the fragment if it was shorter than 500 bp. Besides, they were subjected to the software MetaGeneMark (version 3.3: http://exon.gatech.edu/GeneMark/metagenome/Prediction) to predict the open reading frames (ORFs). Linclust software (https://www.nature.com/articles/s41467-018-04964-5) was used for genetic clustering and eliminating redundancy. The clean reads without redundancy were mapped to the gene catalog using bbmap software, and then, the relative abundances of the genes were calculated. Alpha diversity was evaluated by the Simpson index, the Chao index, the Shannon index, and the Ace index. Beta diversity among groups was generated based on Anosim (analysis of similarities) algorithms and reported according to principal coordinate analysis (PCA). The predicted ORFs were compared with the Kyoto Encyclopedia of Genes and Genomes (KEGG) pathways. The linear discriminant analysis effect size (LEfSe) was used to differentiate the fecal microbial features and KEGG pathways (the default LDA score was 3).

### Metabolomic analysis

The extracting solution [methyl alcohol, acetonitrile, and ultrapure water, = 2:2:1 (v/v)] was added to the colon contents with a vortex for 30 s, followed by grinding for 4 min at 35 Hz and ultrasonication on ice for 5 min, all three times. The samples were stationary at −40°C for 1 h and ultracentrifuged at 4°C and 12,000 rpm for 15 mins. Liquid chromatography-tandem mass spectrometry (LC-MS/MS) analysis of the supernates was conducted with an ultra-performance liquid chromatograph (Vanquish, Thermo Fisher Scientific), a UPLC BEH Amide column (2.1 × 100 mm, 1.7 μm), and a Q Exactive HFX mass spectrometer (Orbitrap MS, Thermo). The autosampler temperature was 4°C, and the injection volume was 3 μL. Gradient elution of the analytes was performed with 25 mmol/L ammonium acetate and 25 mmol/L ammonia water (pH = 9.75) in water and acetonitrile. The spray voltages were 3.6 and 3.2 kV in positive and negative ion modes, respectively, with the capillary temperature at 350°C. The full scan was performed at a resolution of 60,000. The collisional energy was 10/30/60 under the NEC mode. PCA and orthogonal projections to latent structure discriminant analysis (OPLS-DA) were performed using SIMCA14 software (V15.0.2, Sartorius Stedim Data Analytics AB, Umea, Sweden) with the LC-MS/MS data. The metabolites with the first principal component of variable importance projection (VIP) values higher than 1.0 and p value < 0.05 by Student's t-test were considered to be the significantly different metabolites. A metabolomic analysis was conducted through Guangzhou Yike Biotechnology Co., Ltd. (Guangzhou, China).

### Correlation analysis of the gut microbiota and metabolites

The correlation between the gut microbiome and metabolites was evaluated by Pearson correlation analysis. *P*-values of 0.05 were considered statistically significant. A heatmap was used to show the correlation analysis.

### Statistical analysis

Data are presented as means ± standard deviations. Differences between groups were analyzed by one-way analysis of variance (ANOVA). In the multi-comparison, the least significant difference (LSD) was used. The level of statistical significance was set at a *P*-value of 0.05. All datasets were analyzed using SPSS 25 (SPSS, Chicago, IL, United States).

## Results

### Hemicellulose reduced fasting blood glucose but did not exert weight-loss effects in db/db mice

**Figure 1:**
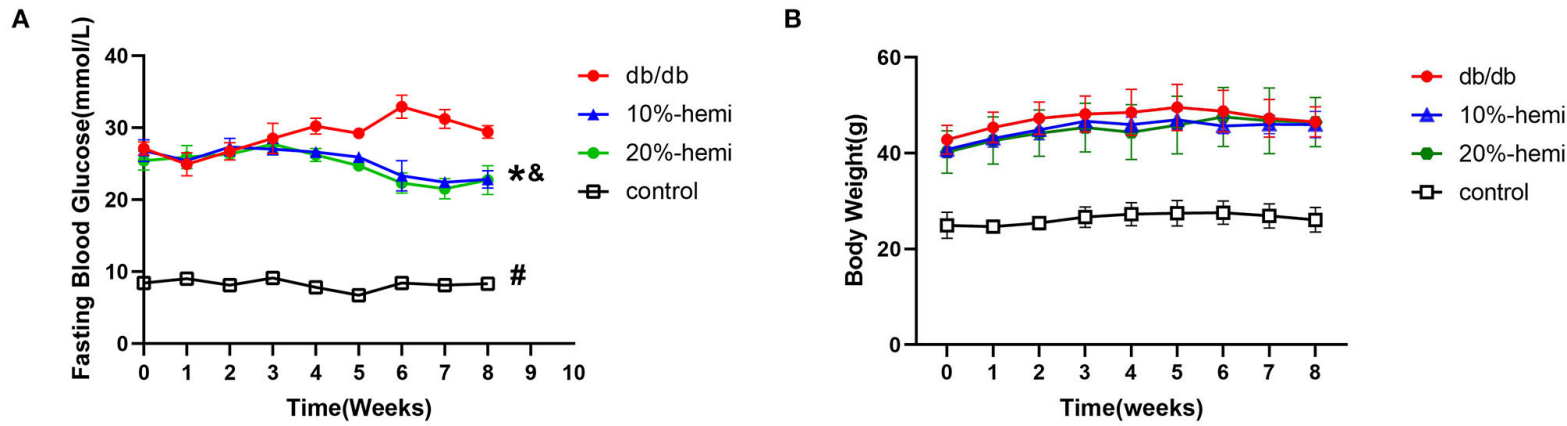
Fasting blood glucose **(A)** and body weight **(B)** of the mice for the four groups (# db/db Vs. control, *p* < 0.05; *10%-hemi Vs. db/db, *p* < 0.05; & 20%-hemi Vs. db/db, *p* < 0.05).

Fasting blood glucose was significantly higher in db/db mice when compared to the control mice during the whole experiment. Fasting blood glucose was gradually decreased by both 10% and 20% hemicellulose supplementation and was significantly lower than that of db/db mice at week 8. No significant differences were found between these two groups (Figure 1A). However, hemicellulose showed an anti-hyperglycemic effect but did not exert a weight-loss effect (Figure 1B).

### Hemicellulose alleviated the impairment of the gut barrier and reduced the serum LPS in db/db mice

**Figure 2:**
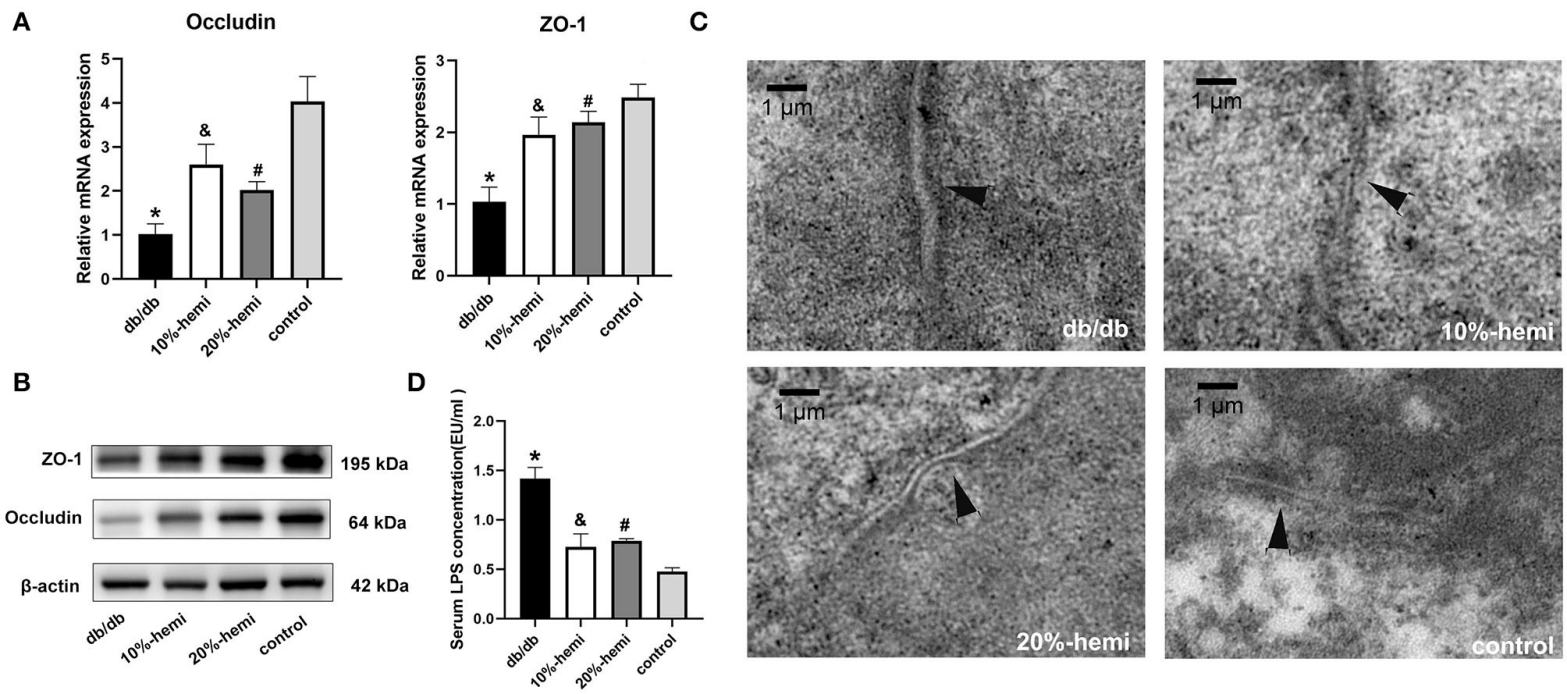
TJ protein expression, intercellular gap of the colonic epithelium, and serum LPS levels for the four groups. **(A**, **B)** mRNA and protein expressions of occludin and ZO-1 of the colonic epithelium for the four groups (* db/db Vs. control, *p* < 0.01; &10%-hemi Vs. db/db, *p* < 0.01; # 20%-hemi Vs. db/db, *p* < 0.01). **(C)** intercellular gap of the colonic epithelium for the four groups showed by TEM (scale bar =1 μm). **(D)** serum LPS levels for the four groups. (*db/db Vs. control, *p* < 0.01; &10%-hemi Vs. db/db, *p* < 0.01; # 20%-hemi Vs. db/db, *p* < 0.01).

The mRNA and protein expressions of occludin and ZO-1, which were TJ proteins, were lower in the db/db mice than those in the control mice. The mRNA and protein expressions of occludin and ZO-1 were restored in mice fed by a diet supplemented with 10% and 20% hemicellulose. Furthermore, no differences were found between the 10% and 20% hemicellulose groups (Figures 2A, B).

TEM results showed that a wider intercellular gap in the colonic epithelium was observed in db/db mice when compared to the control mice. The gaps were significantly narrower in mice-fed diets with 10% and 20% hemicellulose supplementation (Figure 2C). Moreover, serum LPS levels were markedly increased in the db/db group compared to those in the control group. With supplementation of 10% or 20% hemicellulose, serum LPS levels dropped significantly (Figure 2D). No significant differences were found between the 10% and 20% hemicellulose groups.

### Hemicellulose changes the gut microbiota in db/db mice

**Figure 3:**
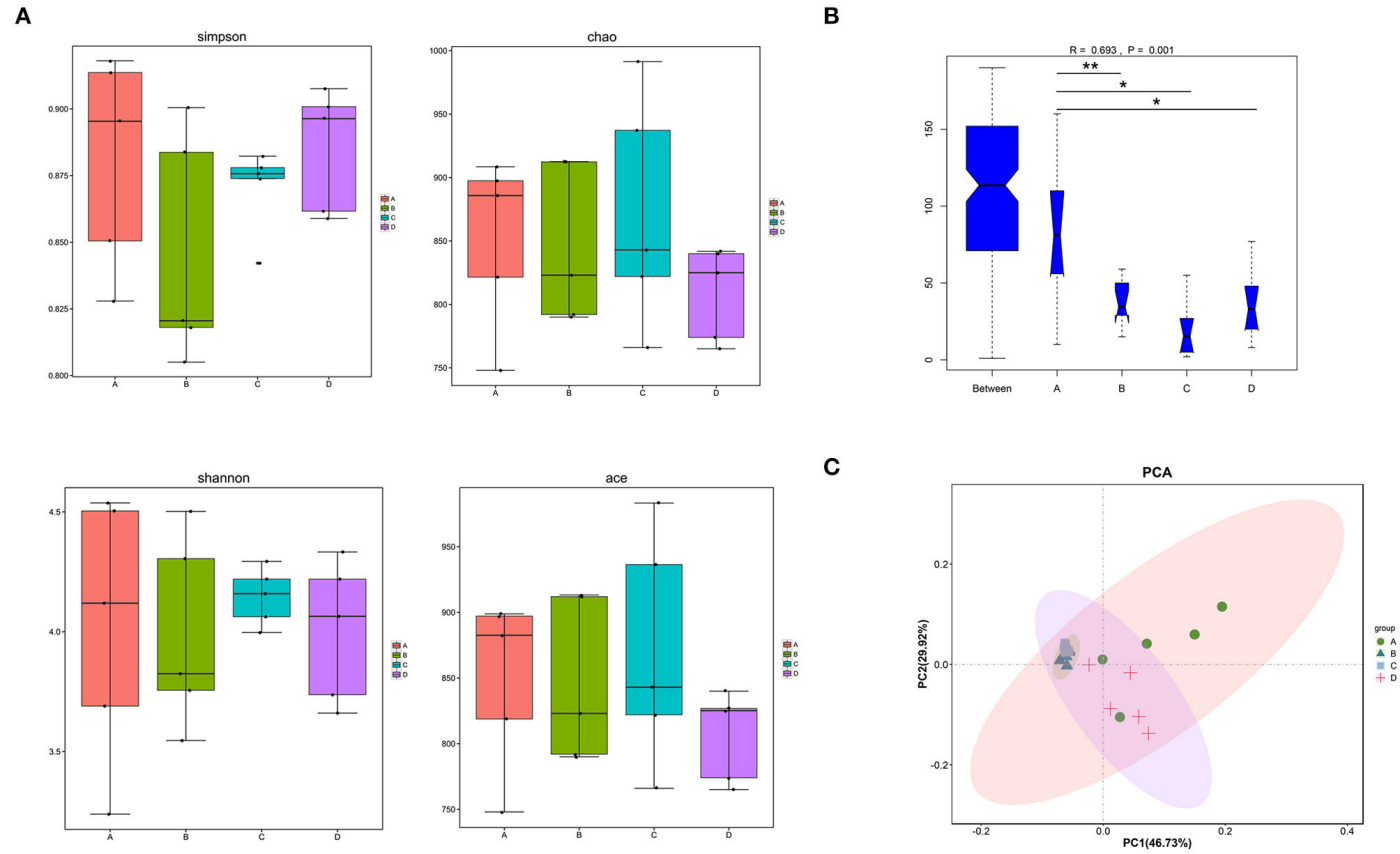
Differences in the gut microbiota among the four groups. **(A)** alpha diversities. **(B)** beta diversities (*p < 0.05; **p < 0.01). **(C)** PCA at the genus level.

Alpha diversities showed no significant discrepancies among the four groups in gut microbiota at the genus level (Figure 3A). However, beta-diversity diversities revealed significant differences among the four groups at genus levels (ANOSIM, *p* = 0.001; Figure 3B). To evaluate the effects of hemicellulose on the microbial community, PCA plots based on distance were performed (Figure 3C). At the genus level, it showed that the degree of clustering of mice in groups B, C, and D was high, whereas the degree of clustering between groups A and these three groups was low, which indicated that the hemicellulose treatment partially abrogated the alteration of microbiota by T2DM.

**Figure 4:**
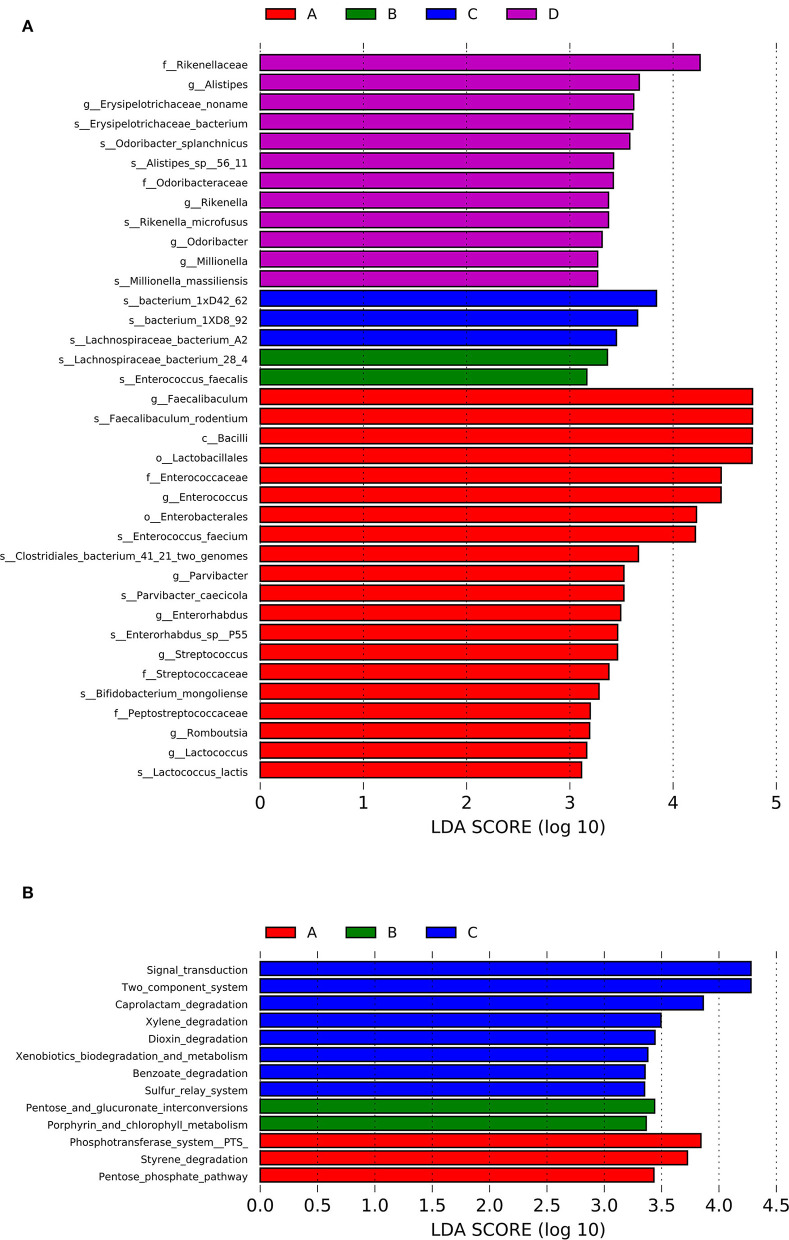
LEfSe analysis of the differential relative abundances of the gut microbiota **(A)** and the KEGG enrichment pathways **(B)** for the four groups.

LEfSe analysis was conducted to identify the differential relative abundances of the microbiota (LDA score > 3.0, *P* < 0.05; Figure 4A) and showed significant differences among the four groups. Class *Bacilli* and genera *Faecalibaculum, Enterococcus*, and *Parvibacter* were enriched in the A group. In the D group, the families *Rikenellaceae* and *Odoribactereae* and the genera *Alistipes, Erysipelotrichaceae, Odoribacter, Millionella*, and *Rikenella* were enriched. In B and C groups, the genera *Lachnospiraceae_noname* and *Enterococcus* were enriched. Based on the LEfSe analysis, a detailed analysis of 14 genera with differential relative abundances is shown in Table 3.

**Table 3:** Detailed analysis of the genera with differential relative abundances.

**Genus**	**D**	**B**	**C**
*Bifidobacterium*	–	↓	↓
*Lactococcus*	↓	↓	↓
*Faecalibaculum*	↓	↓	↓
*Enterococcus*	↓	↓	↓
*Romboutsia*	↓	↓	↓
*Enterorhabdus*	↓	↓	↓
*Parvibacter*	↓	↓	↓
*Clostridiales_noname*	↓	↓	↓
*Streptococcus*	↓	↓	↓
*Lachnospiraceae_noname*	↓	–	–
*Alistipes*	↑	–	–
*Millionella*	↑	–	–
*Rikenella*	↑	–	–
*Odoribacter*	↑	–	–

The relative abundances compared to group A. ↑increased relative abundances; ↓ decreased relative abundances; –no differences.

Based on the Kyoto Encyclopedia of Genes and Genomes (KEGG) database, the functional capabilities of microbiota were estimated. The KEGG enrichment pathways were analyzed by LEfSe analysis (LDA score>3.0; *P* < 0.05; Figure 4B). Styrene degradation, pentose phosphate, and phosphotransferase system pathways were enriched in the A group. The entose and glucuronate interconversions and Porphyrin and chlorophyll metabolism pathways were enriched in B group. Dioxin degradation, Caprolactam degradation, Xylene degradation, Benzoate degradation, Sulfur relay system, and Two component system pathways were enriched in C group.

### Hemicellulose affects the gut metabolome

**Figure 5:**
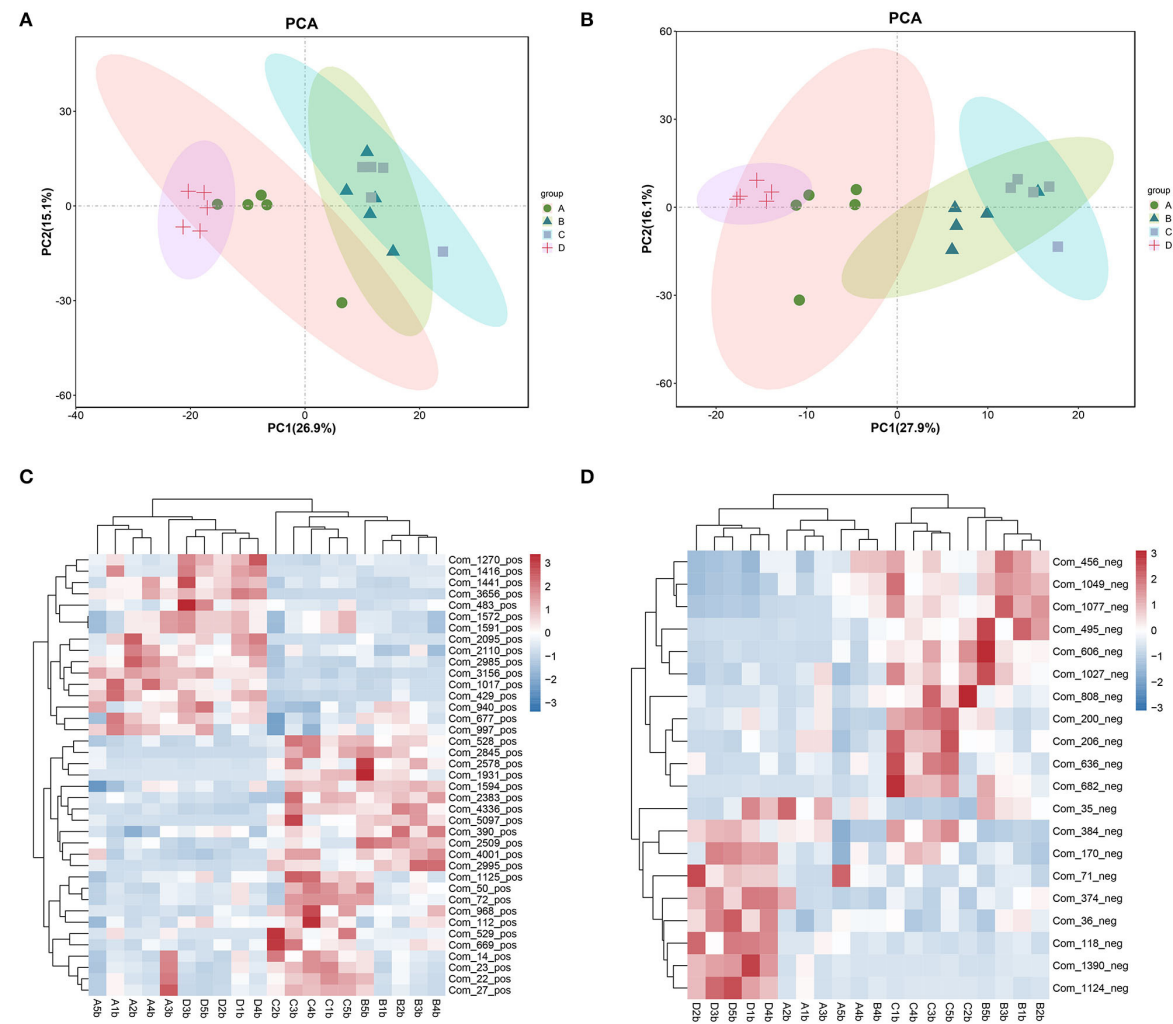
PCA showing the metabolomic profiles of the fecal contents and a heatmaps showing the significantly different metabolites among the four groups. **(A, C)** positive ion mode. **(B, D)** negative ion mode.

The metabolomic profiles of the colonic contents from groups A–D of mice were compared using PCA to visually separate the different groups. It was shown that, among the four groups, the metabolomic profiles showed significant differences (Figures 5A, B). However, groups B and C were more similar, especially in the positive ion mode (Figure 5B). In the positive ion mode, there were 39 significantly different metabolites (threshold VIP > 1.0, *p* < 0.05; Figure 5C). In the negative ion mode, there were 20 significantly different metabolites (threshold VIP > 1.0, *p* < 0.05; Figure 5D). The substances matching the code name are shown in Supplementary Table 1.

### Correlation between the gut microbiota and metabolome

**Figure 6:**
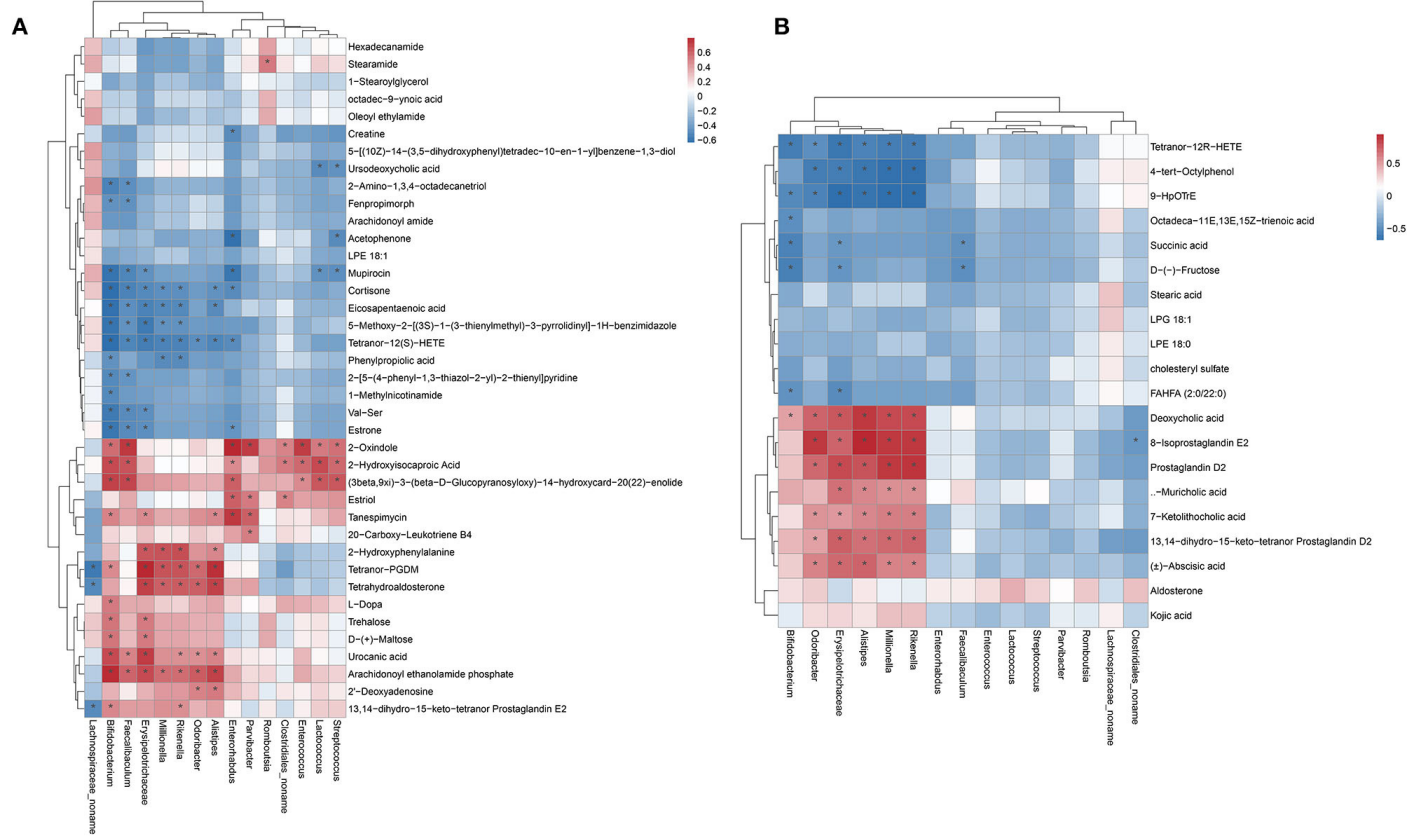
Heatmap showing the Pearson correlation analysis between the gut microbiota and metabolites. **(A)** positive ion mode. **(B)** negative ion mode.

The correlation between the gut microbiota and metabolome was further analyzed in db/db mice after hemicellulose treatment (positive ion mode, Figure 6A; negative ion mode, Figure 6B). Particularly, genera *Lachnospiraceae_noname* and *Enterococcus* were enriched in the B and C groups. Tetranor-PGDM, tetrahydroaldosterone, and 13,14-dihydro-15-keto-tetranor Prostaglandin E2 were all found to be negatively related to the genus *Lachnospiraceae_noname*. 2-Oxindole, 2-Hydroxyisocaproic Acid, and (3beta, 9xi) -3-(beta-D-Glucopyranosyloxy) -14-hydroxycard-20(22) -enolide was positively correlated with Genus *Enterococcus*. Moreover, succinate was negatively correlated with the genera *Bifidobacterium, Erysipelatoclostridium*, and *Faecalibaculum*. Ursodeoxycholic acid (UDCA) was found to be negatively associated with the bacteria genus *Lactococcus* and *Streptococcus*.

### Hemicellulose reduced colonic inflammatory cytokine expression in db/db mice

**Figure 7:**
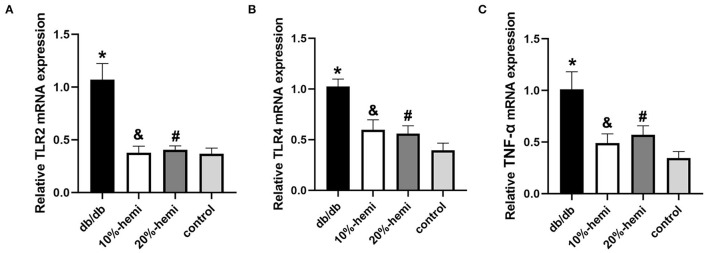
Colonic inflammatory cytokine expression for the four groups (* db/db Vs. control, *p* < 0.01; &10%-hemi Vs. db/db, *p* < 0.01; # 20%-hemi Vs. db/db, *p* < 0.01).

To demonstrate the effects of hemicellulose on colonic inflammation, we used qPCR to compare the mRNA expressions of TLR2, TLR4, and TNF-α in the colon among the four groups. The expressions of TLR2, TLR4, and TNF-α were significantly higher in the db/db group than in the control group. In addition, hemicellulose supplementation significantly reduced the expressions of TLR2, TLR4, and TNF-α in db/db mice (Figure 7). No significant differences were found between the 10% and 20% hemicellulose groups.

## Discussion

T2DM is a complex metabolic disease, and chronic systemic inflammation is considered one of its crucial physiopathologic bases. On the other hand, with the rapid development of sequencing and spectrometry technology, the role of gut microbiota and its metabolites in disease progression, especially in metabolic disease, has drawn much attention. Dysbiosis in T2DM was illustrated in our and other previous studies (Qin et al., 2012; Zhang et al., 2019). Dysbiosis compromises the integrity of the gut barrier, allowing intestinal bacteria and their PAMPs to enter the bloodstream. It promoted systemic inflammation and regulated host metabolism (Nicholson et al., 2012; Wei et al., 2020). Basis on this, supplementation with probiotics or prebiotics to help maintain the integrity of the gut barrier appears to be a safe and potential treatment for T2DM. Hemicellulose, one kind of dietary fiber, was a potential prebiotic.

In this study, we found that, although body weight did not differ significantly, better glucose control was achieved with hemicellulose supplementation (Figure 1). Moreover, we evaluated the impact of hemicellulose on the gut barrier. First, impairment of the gut barrier in T2DM was observed; TJ proteins ZO-1 and occludin expressions were decreased, the intercellular gap deteriorated, and serum LPS was remarkably higher in db/db mice (Figure 2). It was consistent with the previous studies (Zhang et al., 2019; Huang et al., 2021; Yuan et al., 2021). Gut bacteria and their PAMPs, such as LPS, could enter the bloodstream *via* the leaky gut. It served as one of the bases for chronic systemic inflammation in T2DM. As a result, the leaky gut contributed to the progression of T2DM, and LPS served as a blood indicator for gut hyper-permeability and systemic inflammation (Lee et al., 2020). Hemicellulose restored the ZO-1 and occludin expressions and the deteriorated intercellular gap in colonic epithelium of db/db mice, and consequently lowered the systemic LPS levels (Figure 2). These results indicated that hemicellulose alleviated the impaired gut barrier and helped to control hyperglycemia by lowering systemic inflammation.

One of the important cause of the disruption of the gut barrier was the changes in the microbiota (Wei et al., 2020). An illustration of how hemicellulose modulates the gut microbiota may shed light on its gut barrier-repairing effects. Therefore, we further analyzed the microbiota profiles of the mice in the four groups. Though there were no significant differences in alpha diversities among the four groups, beta diversities showed significant differences. And the similarities among B, C, and D groups were high and A group was different from the other three groups (Figure 3). It indicated that, though hemicellulose could not modulate the gut microbiota's biodiversity, it could reshape its composition. As was shown in the LEfSe analysis, the genera *Faecalibaculum* and *Enterorhabdus* were enriched in the db/db mice rather than the control mice, which were decreased after hemicellulose treatment in db/db mice (Figure 4A, Table 3). It was consistent with the previous study, which showed that genus *Enterorhabdus* was enriched in prediabetic adults and decreased by prebiotic xylooligosaccharide (Yang et al., 2015). Moreover, xylooligosaccharide also showed an anti-hyperglycemic effect, indicating that the genus *Enterorhabdus* might participate in the anti-hyperglycemic effect of the prebiotics, including hemicellulose. On the other hand, it was shown that the genus *Faecalibaculum* was enriched in cerebral ischemic stroke and associated with increased intestinal permeability (Chen et al., 2019). Relative redundances of genus *Faecalibaculum* decreased after hemicellulose supplementation, indicating that genus *Faecalibaculum* might play an important role in the effect of hemicellulose on repairing the gut barrier.

Subsequently, based on the KEGG database, the functional capabilities of microbiota were estimated (Figure 4B). Particularly, the phosphotransferase system pathway was enriched in the A group rather than in the other three groups and was associated with obesity (Ni et al., 2020). The dioxin degradation pathway was enriched in the C group. A previous study showed that dioxin increased markers of intestinal inflammation. (Petriello et al., 2018) Enrichment in the dioxin degradation pathway might decrease intestinal inflammation and help maintain gut integrity. They might clue in on the underlying mechanism of the anti-hyperglycemic effect and the reparation of the gut barrier by hemicellulose.

The gut microbiota-derived metabolites play critical roles in the maintenance of gut health and the progression of the disease, including maintaining the integrity of the gut barrier (Sugimura et al., 2021; Wu et al., 2022). We next analyzed the metabolomic profiles of the fecal contents. The metabolomic profiles of the four groups differed significantly. However, the B and C groups were more similar, indicating that hemicellulose had a specific effect on the gut metabolome (Figures 5A, B). We found that succinate and UDCA were particularly increased after hemicellulose supplementation (Figures 5C, D). It was consistent with the previous studies in that it showed that increased succinate in the gut could reduce hyperglycemia and that both succinate and UDCA could protect the gut barrier. The mechanisms involved in these protective effects include modulating intestinal inflammation, ameliorating oxidative stress, and promoting enterocyte migration (Bernardes-Silva et al., 2004; Golden et al., 2018; Li et al., 2019; Wang et al., 2019). Succinate and UDCA might be the key metabolites underlying the mechanism of the treatment effects of hemicellulose on the impaired gut barrier and hyperglycemia in T2DM.

Some key microbes found in the human gut produce critical metabolic bioactive signaling molecules that aid in the maintenance of health and the onset of disease (Wang and Zhao, 2018). Thus, we further investigated the correlation between gut metabolites and microbiota (Figure 6). We found that succinate was negatively correlated with the genera *Bifidobacterium, Erysipelatoclostridium*, and *Faecalibaculum*. UDCA was negatively correlated to genus *Lactococcus* and *Streptococcus*. Specifically, the relative abundances of the genera *Bifidobacterium, Faecalibaculum, Lactococcus*, and *Streptococcus* decreased, and succinate and UDCA increased after hemicellulose supplementation. It indicated that they might also be the key bacteria underlying the mechanism of the treatment effects of hemicellulose on the impaired gut barrier and hyperglycemia in T2DM.

The intestinal bacterial dysbiosis contributed to intestinal barrier impairments, and one of the most important underlying mechanisms was believed to be its role in modulating gut inflammation (Yang et al., 2021). As shown above, the functional capabilities of the gut microbiota from the db/db mice treated with hemicellulose were enriched in the dioxin degradation pathway that ameliorated intestinal inflammation (Figure 4B). Additionally, the crucial metabolites found in this study, namely succinate and UDCA, were also found to be able to exert anti-inflammatory actions in the colon (Ward et al., 2017; Li et al., 2019). Based on this, we evaluated the inflammation levels in the colon for the four groups. The serum LPS levels were elevated in db/db mice and lowered by hemicellulose. The TLRs could recognize a variety of microbial structural components known as PAMPs, including LPS. Hence, we detected TLR/TNF-α signaling activities and found that they were upregulated in db/db mice and decreased by hemicellulose supplementation (Figure 7). Hemicellulose supplementation might protect the gut barrier by modulating intestinal inflammation *via* the gut microbiota and its metabolites.

Despite the strengths, there were some limitations to this study. Through the use of metagenomic sequencing and liquid chromatography-mass spectrometry, we identified specific genera of gut microbiota and gut metabolites that may contribute to the beneficial effects of hemicellulose on gut barrier integrity and hyperglycemia in T2DM. However, further validation by supplementation or elimination of the specific bacteria or metabolite is needed. Besides, we found some key correlations between the gut microbiota and metabolites, but further validation of whether the changes in metabolites are attributed to the indicated bacteria is needed.

In conclusion, this study provides evidence for the beneficial effects of hemicellulose on hyperglycemia and gut permeability in mice model with T2DM. Hemicellulose supplementation was also found to modulate gut microbiota composition and metabolome profiles as well as to decrease colonic and systemic inflammation. These findings suggest that hemicellulose may represent a novel therapeutic option for T2DM through its potential to target multiple pathophysiological mechanisms. More research is required to confirm the role of the specific bacteria or metabolite in the hemicellulose treatment effect.

## Data availability statement

The raw sequence data reported in this paper have been deposited in the Genome Sequence Archive (Genomics, Proteomics & Bioinformatics 2021) in National Genomics Data Center (Nucleic Acids Res 2022), China National Center for Bioinformation/Beijing Institute of Genomics, Chinese Academy of Sciences. Accession number is CRA009556. It is publicly accessible at https://ngdc.cncb.ac.cn/gsa. The raw liquid chromatography-mass spectrometry data reported in this paper have been deposited in the Open Archive for Miscellaneous Data, Beijing Institute of Genomics, Chinese Academy of Sciences. Accession number is OMIX002807. The dataset is publicly accessible at https://ngdc.cncb.ac.cn/databasecommons/database/id/7416.

## Ethics statement

The animal study was reviewed and approved by the animal study protocol was approved by Animal Ethics Committee of Sun Yat-Sen University (permit code: SYSU-IACUC-2021-000391).

## Author contributions

HL: data curation, formal analysis, investigation, methodology, software, and validation. JHX: data curation, investigation, methodology, software, visualization, and writing—review and editing. CWY: investigation and project administration. QKC: conceptualization, funding acquisition, and validation. JYL: conceptualization, formal analysis, funding acquisition, validation, writing—original draft, and writing—review and editing.

## References

[B1] Bernardes-Silva C. F. Damião A. O. M. C. Sipahi A. M. Laurindo F. R. M. Iriya K. Lopasso F. P.. (2004). Ursodeoxycholic acid ameliorates experimental ileitis counteracting intestinal barrier dysfunction and oxidative stress. Dig. Dis. Sci. 49, 1569–74. 10.1023/B:DDAS.0000043365.39251.6e 15573906

[B2] Chen R. Wu P. Cai Z. Fang Y. Zhou H. Lasanajak Y.. (2019). Puerariae Lobatae Radix with chuanxiong Rhizoma for treatment of cerebral ischemic stroke by remodeling gut microbiota to regulate the brain-gut barriers. J. Nutr. Biochem. 65, 101–114. 10.1016/j.jnutbio.2018.12.004 30710886

[B3] Cheng D. Xu J.-H. Li J.-Y. Wang S.-Y. Wu T.-F. Chen Q.-K.. (2018). Butyrate ameliorated-NLRC3 protects the intestinal barrier in a GPR43-dependent manner. Exp. Cell Res. 368, 101–110. 10.1016/j.yexcr.2018.04.018 29689277

[B4] de Vos W. M. Tilg H. Van Hul M. Cani P. D. (2022). Gut microbiome and health: mechanistic insights. Gut. 71, 1020–1032. 10.1136/gutjnl-2021-326789 35105664 PMC8995832

[B5] Desai M. S. Seekatz A. M. Koropatkin N. M. Kamada N. Hickey C. A. Wolter M.. (2016). A dietary fiber-deprived gut microbiota degrades the colonic mucus barrier and enhances pathogen susceptibility. Cell. 167, 1339–1353. e21. 10.1016/j.cell.2016.10.043 27863247 PMC5131798

[B6] Donath M. Y. Shoelson S. E. (2011). Type 2 diabetes as an inflammatory disease. Nat. Rev. Immunol. 11, 98–107. 10.1038/nri2925 21233852

[B7] Federation I. D. (2019). IDF Atlas-Nine Edition. IDF Diabetes atlas 9th edition 2019. Available online at: www.diabetesatlas.org (accessed November 18, 2019).

[B8] Ghosh S. S. Wang J. Yannie P. J. Ghosh S. (2020). Intestinal barrier dysfunction, lps translocation, and disease development. J. Endocr. Soc. 4, bvz039. 10.1210/jendso/bvz039 32099951 PMC7033038

[B9] Golden J. M. Escobar O. H. Nguyen M. V. L. Mallicote M. U. Kavarian P. Frey M. R.. (2018). Ursodeoxycholic acid protects against intestinal barrier breakdown by promoting enterocyte migration *via* EGFR- and COX-2-dependent mechanisms. Am. J. Physiol. Gastrointest. Liver Physiol. 315, G259–G271. 10.1152/ajpgi.00354.2017 29672156 PMC6139640

[B10] Guo Y. Yu Y. Li H. Ding X. Li X. Jing X.. (2021). Inulin supplementation ameliorates hyperuricemia and modulates gut microbiota in Uox-knockout mice. Eur. J. Nutr. 60, 2217–2230. 10.1007/s00394-020-02414-x 33104864 PMC8137640

[B11] Huang J. Guan B. Lin L. Wang Y. (2021). Improvement of intestinal barrier function, gut microbiota, and metabolic endotoxemia in type 2 diabetes rats by curcumin. Bioengineered. 12, 11947–11958. 10.1080/21655979.2021.2009322 34818970 PMC8810160

[B12] Lee C. J. Sears C. L. Maruthur N. (2020). Gut microbiome and its role in obesity and insulin resistance. Ann. N. Y. Acad. Sci. 1461, 37–52. 10.1111/nyas.14107 31087391

[B13] Li X. Mao M. Zhang Y. Yu K. Zhu W. (2019). Succinate modulates intestinal barrier function and inflammation response in pigs. Biomolecules. 9, 486. 10.3390/biom9090486 31540325 PMC6770553

[B14] Ni Y. Yu G. Chen H. Deng Y. Wells P. M. Steves C. J.. (2020). M2IA: a web server for microbiome and metabolome integrative analysis. Bioinformatics. 36, 3493–3498. 10.1093/bioinformatics/btaa188 32176258

[B15] Nicholson J. K. Holmes E. Kinross J. Burcelin R. Gibson G. Jia W. Pettersson S. (2012). Host-gut microbiota metabolic interactions. Science. 336, 1262–7. 10.1126/science.1223813 22674330

[B16] Petriello M. C. Hoffman J. B. Vsevolozhskaya O. Morris A. J. Hennig B. (2018). Dioxin-like PCB 126 increases intestinal inflammation and disrupts gut microbiota and metabolic homeostasis. Environ. Pollut. 242, 1022–1032. 10.1016/j.envpol.2018.07.039 30373033 PMC6211811

[B17] Qin J. Li Y. Cai Z. Li S. Zhu J. Zhang F.. (2012). A metagenome-wide association study of gut microbiota in type 2 diabetes. Nature. 490, 55–60. 10.1038/nature11450 23023125

[B18] Sugimura N. Li Q. Chu E. S. H. Lau H. C. H. Fong W. Liu W.. (2021). *Lactobacillus gallinarum* modulates the gut microbiota and produces anti-cancer metabolites to protect against colorectal tumourigenesis. Gut. 71, 2011–2021. 10.1136/gutjnl-2020-323951 34937766 PMC9484392

[B19] Wang K. Liao M. Zhou N. Bao L. Ma K. Zheng Z.. (2019). Parabacteroides distasonis alleviates obesity and metabolic dysfunctions *via* production of succinate and secondary bile acids. Cell Rep. 26, 222–235. e5. 10.1016/j.celrep.2018.12.028 30605678

[B20] Wang Z. Zhao Y. (2018). Gut microbiota derived metabolites in cardiovascular health and disease. Protein Cell. 9, 416–431. 10.1007/s13238-018-0549-0 29725935 PMC5960473

[B21] Ward J. B. J. Lajczak N. K. Kelly O. B. O'Dwyer A. M. Giddam A. K. Gabhann J. N.. (2017). Ursodeoxycholic acid and lithocholic acid exert anti-inflammatory actions in the colon. Am. J. Physiol. Gastrointest. Liver Physiol. 312, G550–G558. 10.1152/ajpgi.00256.2016 28360029

[B22] Wei L. Yue F. Xing L. Wu S. Shi Y. Li J.. (2020). Constant light exposure alters gut microbiota and promotes the progression of steatohepatitis in high fat diet rats. Front. Microbiol. 11, 1975. 10.3389/fmicb.2020.01975 32973715 PMC7472380

[B23] Wlodarczyk M. Slizewska K. (2021). Efficiency of resistant starch and dextrins as prebiotics: a review of the existing evidence and clinical trials. Nutrients. 13, 3808. 10.3390/nu13113808 34836063 PMC8621223

[B24] Wu Z. Xu Q. Gu S. Chen Y. Lv L. Zheng B.. (2022). *Akkermansia muciniphila Ameliorates Clostridioides* difficile infection in mice by modulating the intestinal microbiome and metabolites. Front. Microbiol. 13, 841920. 10.3389/fmicb.2022.841920 35663882 PMC9159907

[B25] Yang G. Wei J. Liu P. Zhang Q. Tian Y. Hou G.. (2021). Role of the gut microbiota in type 2 diabetes and related diseases. Metab. Clin. Exp. 117, 154712. 10.1016/j.metabol.2021.154712 33497712

[B26] Yang J. Summanen P. H. Henning S. M. Hsu M. Lam H. Huang J.. (2015). Xylooligosaccharide supplementation alters gut bacteria in both healthy and prediabetic adults: a pilot study. Front. Physiol. 6, 216. 10.3389/fphys.2015.00216 26300782 PMC4528259

[B27] Yuan J. H. Xie Q. S. Chen G. C. Huang C. L. Yu T. Chen Q. K.. (2021). Impaired intestinal barrier function in type 2 diabetic patients measured by serum LPS, Zonulin, and IFABJ. Diabetes Complications. 35, 107766. 10.1016/j.jdiacomp.2020.107766 33168395

[B28] Zhang W. Xu J.-H. Yu T. Chen Q.-K. (2019). Effects of berberine and metformin on intestinal inflammation and gut microbiome composition in db/db mice. Biomed. Pharmacother. 118, 109131. 10.1016/j.biopha.2019.109131 31545226

[B29] Zou J. Chassaing B. Singh V. Pellizzon M. Ricci M. Fythe M. D.. (2018). Fiber-mediated nourishment of gut microbiota protects against diet-induced obesity by restoring IL-22-mediated colonic health. Cell Host Microbe. 23, 41–53. e4. 10.1016/j.chom.2017.11.003 29276170 PMC6005180

