# Supplementary material for: Effects of hemicellulose on intestinal mucosal barrier integrity, gut microbiota, and metabolomics in a mouse model of type 2 diabetes mellitus
Source: Front Microbiol. 2023 Feb 6;14:1096471. doi: 10.3389/fmicb.2023.1096471 (PMC9942597; doi:10.3389/fmicb.2023.1096471)
Supplement: Supplementary file 1 [file Table_1.DOCX]

Supplementary Table.

Supplementary Table 1. The substances matching the code name.

| negative ion mode | | positive ion mode | |
| --- | --- | --- | --- |
| Com_1390_neg | 8-Isoprostaglandin E2 | Com_50_pos | Fenpropimorph |
| Com_1124_neg | Prostaglandin D2 | Com_23_pos | Oleoyl ethylamide |
| Com_118_neg | Deoxycholic acid | Com_27_pos | Stearamide |
| Com_1049_neg | 9-HpOTrE | Com_22_pos | Hexadecanamide |
| Com_1077_neg | Tetranor-12R-HETE | Com_14_pos | octadec-9-ynoic acid |
| Com_200_neg | Stearic acid | Com_72_pos | Arachidonoyl amide |
| Com_456_neg | 4-tert-Octylphenol | Com_390_pos | Creatine |
| Com_374_neg | (±)-Abscisic acid | Com_528_pos | Mupirocin |
| Com_36_neg | 13,14-dihydro-15-keto-tetranor Prostaglandin D2 | Com_429_pos | Tanespimycin |
| Com_495_neg | Succinic acid | Com_112_pos | Ursodeoxycholic acid |
| Com_206_neg | cholesteryl sulfate | Com_1017_pos | 2-Oxindole |
| Com_384_neg | Kojic acid | Com_968_pos | 2-Amino-1,3,4-octadecanetriol |
| Com_170_neg | 7-Ketolithocholic acid | Com_529_pos | 1-Stearoylglycerol |
| Com_682_neg | Octadeca-11E,13E,15Z-trienoic acid | Com_2509_pos | Phenylpropiolic acid |
| Com_808_neg | LPE 18:0 | Com_669_pos | LPE 18:1 |
| Com_606_neg | D-(-)-Fructose | Com_2845_pos | Cortisone |
| Com_636_neg | LPG 18:1 | Com_1931_pos | 2-[5-(4-phenyl-1,3-thiazol-2-yl)-2-thienyl]pyridine |
| Com_1027_neg | FAHFA (2:0/22:0) | Com_1594_pos | Acetophenone |
| Com_71_neg | β-Muricholic acid | Com_3156_pos | (3beta,9xi)-3-(beta-D-Glucopyranosyloxy)-14-hydroxycard-20(22)-enolide |
| Com_35_neg | Aldosterone | Com_2578_pos | 1-Methylnicotinamide |
|  |  | Com_1441_pos | Urocanic acid |
|  |  | Com_2995_pos | Val-Ser |
|  |  | Com_1125_pos | 5-[(10Z)-14-(3,5-dihydroxyphenyl)tetradec-10-en-1-yl]benzene-1,3-diol |
|  |  | Com_4336_pos | Tetranor-12(S)-HETE |
|  |  | Com_3656_pos | Arachidonoyl ethanolamide phosphate |
|  |  | Com_1416_pos | Tetrahydroaldosterone |
|  |  | Com_997_pos | Estriol |
|  |  | Com_4001_pos | 5-Methoxy-2-[(3S)-1-(3-thienylmethyl)-3-pyrrolidinyl]-1H-benzimidazole |
|  |  | Com_940_pos | 13,14-dihydro-15-keto-tetranor Prostaglandin E2 |
|  |  | Com_2095_pos | 2'-Deoxyadenosine |
|  |  | Com_5097_pos | Estrone |
|  |  | Com_1270_pos | Tetranor-PGDM |
|  |  | Com_2110_pos | L-Dopa |
|  |  | Com_1572_pos | Trehalose |
|  |  | Com_483_pos | 2-Hydroxyphenylalanine |
|  |  | Com_2383_pos | Eicosapentaenoic acid |
|  |  | Com_2985_pos | 2-Hydroxyisocaproic Acid |
|  |  | Com_677_pos | 20-Carboxy-Leukotriene B4 |
|  |  | Com_50_pos | Fenpropimorph |
